# Left Ventricular Assist Device Implantation Under Argatroban Anticoagulation in Heparin-Induced Thrombocytopenia: A Literature Review and Clinical Case Presentation

**DOI:** 10.3390/jcm14124083

**Published:** 2025-06-09

**Authors:** Juš Kšela, Jan Kafol, Miha Kerin, Dejan Pirc, Robert Novak, Tomaz Goslar

**Affiliations:** 1 Department of Cardiovascular Surgery, University Medical Centre Ljubljana, 1000 Ljubljana, Slovenia; jus.ksela@kclj.si (J.K.); robert.novak@kclj.si (R.N.); 2 Faculty of Medicine, University of Ljubljana, 1000 Ljubljana, Slovenia; jan.kafol@kclj.si (J.K.); miha.kerin8@gmail.com (M.K.); 3 Department of Vascular Diseases, University Medical Centre Ljubljana, 1000 Ljubljana, Slovenia; 4 Department of Anesthesiology and Surgical Intensive Care, University Medical Centre Ljubljana, 1000 Ljubljana, Slovenia; dejan.pirc@kclj.si; 5 Department of Intensive Internal Medicine, University Medical Centre Ljubljana, 1000 Ljubljana, Slovenia

**Keywords:** heart failure, left ventricular assist device, heparin-induced thrombocytopenia, argatroban, anticoagulation, heart transplantation, cardiac surgery

## Abstract

This review provides an in-depth analysis of argatroban as an alternative anticoagulant in cardiac surgery, with a focus on its use in patients with heparin-induced thrombocytopenia (HIT). We examine argatroban’s pharmacokinetics and dosing regimens and the challenges associated with cosnventional monitoring methods—such as activated clotting time (ACT) and activated partial thromboplastin time (aPTT)—to evaluate its safety and effectiveness in high-risk surgical settings. Drawing on data from multiple case reports and series, our review highlights both the potential benefits and limitations of argatroban, including complications such as clot formation in extracorporeal circulation systems and prolonged postoperative coagulopathy. In addition to the literature review, we present a detailed clinical case of urgent HeartMate 3 left ventricular assist device implantation in a patient with advanced heart failure and active HIT. In this case, despite targeting an ACT above 400 s, intraoperative complications such as clot formation in the heart–lung machine and difficulty achieving hemostasis highlight the need for improved monitoring and dosing protocols. Our findings call for refined anticoagulation strategies and advanced monitoring techniques to optimize argatroban use in cardiac surgery, offering valuable insights for clinicians managing complex scenarios where conventional heparin therapy is contraindicated.

## 1. Introduction

Heart failure represents an enormous clinical and therapeutic challenge and often requires advanced interventions to offer patients a chance of survival. In this situation, left ventricular assist devices (LVADs) have proven to be a crucial bridging solution to transplantation. They prolong the survival of patients while they wait for a suitable donor heart [1]. However, the successful implantation of LVADs can become much more complicated when patients have heparin-induced thrombocytopenia (HIT), a condition characterized by a paradoxical prothrombotic state that requires alternative anticoagulation strategies [2].

In patients with HIT, elective cardiovascular surgery should ideally be postponed. However, when urgent procedures are necessary, alternative anticoagulation strategies can be explored, including bivalirudin, intraoperative heparin following plasma exchange, or a combination of heparin with a potent antiplatelet agent. Despite these options, the supporting evidence remains limited, resulting in only conditional recommendations [3,4].

In this article, we first present a comprehensive review of the literature on argatroban use in cardiac surgery, focusing on its pharmacokinetics, dosing strategies, monitoring challenges, and the management of potential complications in patients with HIT. Building on this foundation, we then illustrate these challenges with a detailed clinical case of urgent LVAD implantation in a patient with advanced heart failure and HIT, where argatroban was employed as the anticoagulant.

## 2. A Review of the Literature on Argatroban Use in Cardiac Surgery

Heparin-induced thrombocytopenia (HIT) is a serious complication caused by the use of heparin. There are two types of HIT, with type 2 being an immune-mediated reaction. It involves the formation of antibodies against the PF4 and heparin complex, which leads to platelet activation and aggregation. Typically, HIT occurs 5–14 days after the initiation of heparin therapy. Clinically, HIT is characterized by a drop in platelet count, either relative (30–50% of baseline) or absolute (50–70 × 10^9^/L). The development of HIT can lead to thromboembolic complications in up to half of patients, even though the risk of bleeding is not usually elevated. Before the diagnosis is made, other possible causes of thrombocytopenia must be excluded [2,5].

To assess the likelihood of HIT, the clinical 4Ts scoring system can be used. The clinical diagnosis should be confirmed by laboratory tests such as PF4 ELISA and serotonin release assay. Treatment is recommended as soon as the clinical 4Ts test reaches a value of 4 or more. Treatment consists of discontinuing heparin treatment and initiating alternative anticoagulation [6].

The 2018 American Society of Hematology (ASH) guidelines recommend alternative anticoagulants for HIT patients with critical illness, a higher risk of bleeding, or urgent need for intervention. Preferred options include argatroban or bivalirudin due to their shorter duration of action [4]. It is important to note that, unlike heparin, these agents do not have a specific reversal agent, so careful monitoring is essential to prevent serious bleeding complications [7]. The successful use of heparin with the concomitant blockade of platelet function with abciximab during cardiac surgery has also been described as an alternative [8].

According to the ASH guidelines, when delaying surgery is not feasible in patients with acute or subacute HIT, bivalirudin is recommended for intraoperative anticoagulation because it has the strongest evidence base compared to other alternatives. In addition to its favorable evidence base, bivalirudin offers a shorter half-life than argatroban and has been associated with lower rates of bleeding—even when compared to heparin—in some studies, making it an attractive option when available. While other strategies—such as intraoperative heparin combined with plasma exchange or potent antiplatelet agents—are mentioned, they lack standardized protocols and are influenced by factors like availability, cost, and clinician experience [4,9].

Argatroban is a synthetic small molecule that acts as a direct thrombin inhibitor. It binds reversibly to thrombin, a key enzyme in the coagulation cascade. Argatroban is not immunogenic and cannot be degraded by serine proteases. It is mainly metabolized by the liver and is, therefore, also suitable for patients with impaired renal function. Its half-life is between 39 and 51 min. Therapeutic argatroban levels are monitored by aPTT or ACT [10]. However, both tests correlate poorly with plasma argatroban concentrations, and further studies are needed to define optimal monitoring approaches [11,12]. Current guidelines restrict the use of argatroban for CPB to patients for whom heparin is contraindicated and where bivalirudin is not a viable option [4].

A review by Martin et al. on the use of argatroban recommended an initial argatroban dose of 0.1–0.3 mg/kg bolus for on-pump cardiac surgery, followed by an infusion of 5–10 µg/kg/min to maintain an ACT above 400. No thrombotic complications have occurred at this dose [3]. In a related report, Green et al. described the successful use of argatroban in a patient with HIT and renal insufficiency undergoing orthotopic heart transplantation. In their case, a loading dose of 200 μg/kg was administered to achieve a target ACT above 400 s, followed by an infusion titrated based on ACT measurements. No thrombotic or hemorrhagic complications were observed, despite delayed aPTT normalization [13].

However, Follis et al. expressed doubts about the recommended ACT above 400, observing blood clots in the HLM oxygenator during cardiac surgery even though the ACT was above 400. Furthermore, after the discontinuation of argatroban, the ACT remained elevated longer than expected based on the half-life of the drug, and significant postoperative bleeding occurred. These results reflect the difficulties and complications we observed in our case. They recommended the use of argatroban in rare cases when other direct thrombin inhibitors are contraindicated and recommended aiming for ACT values of 500–600 with continuous 15 min monitoring intervals [14].

There are few reports of LVAD implantation in HIT patients under argatroban. Hillebrand et al. reported seven clinical cases. In the first case, aiming for an aPTT of 50–60 s, led to thrombotic complications that required LVAD removal. They later modified the approach by aiming for an aPTT of 70–80 s, initially minimizing LVAD stasis time with an anastomosing outflow graft and using cardioplegic arrest for thrombosis inspection. Four of the seven patients had to be revised due to postoperative bleeding [15].

Several studies have reported the delayed normalization of ACT, with thrombotic complications occurring in some cases, even at high ACT values. For instance, Agarwal et al. documented complications at an ACT value of 495, while Mejak et al. observed them at a value of 627 [16,17,18,19]. In contrast, Edwards et al. demonstrated an alternative monitoring strategy during extra-corporal circulation (ECC) in a high-risk HIT patient by using both celite ACT and high-dose thrombin time. Their approach not only provided effective anticoagulation but also suggests that combining these assays may offer a more accurate reflection of argatroban’s activity than ACT alone [20].

## 3. Clinical Case Illustration

A 69-year-old Caucasian man was diagnosed with non-ischemic dilated cardiomyopathy with an initial ejection fraction of 37%. His comorbidities included persistent atrial fibrillation treated with apixaban, arterial hypertension, secondary erythrocytosis, obstructive sleep apnea syndrome, and a history of cerebrovascular insult due to the temporary discontinuation of anticoagulation. Due to his heart failure, he received several levosimendan therapies and underwent intramyocardial stem cell transplantation as part of the CELLpEF 2 trial [21]. Despite these measures, his condition worsened, and his ejection fraction dropped to 25% four years after diagnosis. Due to worsening heart failure and the exhaustion of drug therapies, he was placed on the elective heart transplant list. His condition continued to deteriorate, and six months later, with an ejection fraction of 15, he was urgently admitted to University Medical Centre Ljubljana. On admission, inotropic support with dobutamine and intensified diuretic therapy with furosemide were initiated, and apixaban was switched to continuous unfractionated heparin (UFH). Consequently, his transplant status was upgraded to “urgent”.

Between the 8th and 9th day of hospitalization, a significant drop in platelet count was observed (from 141 × 10^9^/L to 54 × 10^9^/L), which occurred within the expected time frame after starting heparin treatment. Heparin-induced thrombocytopenia (HIT) type II was subsequently confirmed. As bivalirudin was not available, we switched the patient’s anticoagulation to argatroban. As there was no immediate prospect of a suitable heart transplant, we opted for LVAD implantation as a bridge to transplantation. For intraoperative anticoagulation, we adhered to our institution’s protocols, which were informed by the review conducted by Martin et al. [3].

**Figure 1:**
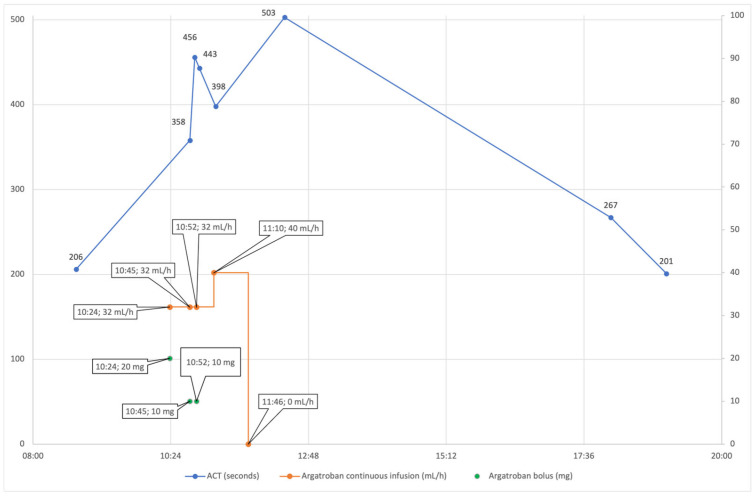
ACT values and argatroban administration on the day of surgery.

**Figure 2:**
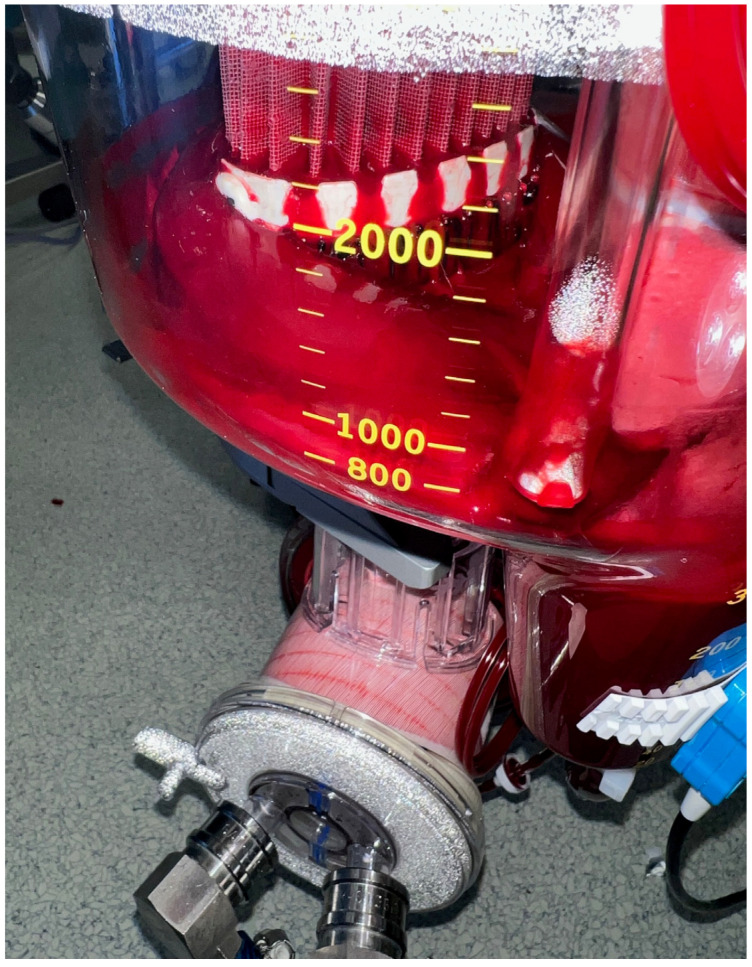
Heart–lung machine blood reservoir with blood clots.

The patient was implanted with an LVAD on the 13th day of hospitalization. Laboratory results before surgery showed an increasing but still slightly low platelet count (134 × 10^9^/L), consistent with recovery from HIT-related thrombocytopenia. Additionally, preoperative labs showed increased fibrinogen (4.7 g/L), increased creatinine (117 μmol/L), slightly increased aspartate and alanine transaminase (0.92 and 0.89 μkat/L), increased total and direct bilirubin (44 and 18 μmol/L), and increased gamma-glutamyltransferase (5.68 μkat/L). The patient received a continuous infusion of dobutamine (3.49 μg/kg/min) and furosemide (1.5 mg/h). The surgical procedure started with a median sternotomy and opening of the pericardium. We used a side-biting (Lambert-Kay) aortic clamp on the ascending aorta to anastomose the LVAD-HeartMate 3 (HM3) outflow graft to the ascending aorta. Sixteen minutes after the surgical incision (INC), a bolus of 20 mg (0.28 mg/kg) argatroban was administered, and a continuous infusion of 32 mg/h (7.4 μg/kg/min) was initiated to achieve an activated clotting time (ACT) of over 400. We used the LivaNova Sorin Stockert S5^TM^ heart–lung machine (HLM), the LivaNova 3T^TM^ heater–cooler system, and the LivaNova Inspire^®^ 8F oxygenator (London, United Kingdom) for ECC. A 22 Fr EOPA cannula was used for aortic cannulation, and a 32/40 Fr dual-stage venous return cannula was used for venous cannulation. ECC preparation included 1200 mL of Ringer’s crystalloid, 250 mL of 20% mannitol, 1 g of methylprednisolone, two units of fresh frozen plasma (241 + 257 mL), and 10 mg of argatroban. Before aortic cannulation (38 min after INC), we checked the ACT, which was 358 (Figure 1), so we administered another 10 mg (0.14 mg/kg) of argatroban. A repeat ACT measurement (in 5 min) was satisfactory, 456, which prompted us to start ECC (45 min after INC). The HM3 pump was implanted using the “sew and cut” technique. After implantation, we started to increase the flow through the HM3 while gradually decreasing the flow through the ECC. Another ACT control (65 min after INC) showed a value of 398, which led to an increase in the argatroban continuous infusion to 40 mg/h (9.3 μg/kg/min). Eighty-three minutes after INC, the perfusionist observed blood clots in the HLM blood reservoir (Figure 2). Fortunately, by this time, the anastomosis and implantation of the HM3 pump had been completed, allowing us to finish the ECC (99 min after INC). Transesophageal echocardiography (TEE) was performed to look for clots in the heart, HM3 pump, and outflow graft, but none were found. The total duration of ECC was 54 min. During ECC, a stable flow through the HLM of 4.3–4.8 L/min was maintained, and the mean arterial pressure remained at 80 mmHg. The patient was not actively cooled, so his body temperature remained between 36.1 and 36.6 °C. The argatroban infusion was discontinued with the completion of ECC. One hundred thirty-seven minutes after INC, the ACT was 503. Since there was no argatroban antidote and the bleeding could not be completely stopped, we decided to postpone the chest closure. We planned to close it after the stabilization of the ACT. During the surgery, the patient received eight units of fresh frozen plasma (two of them during ECC). As the hemoglobin level was satisfactory after the transfusion, the blood from the Cell Saver was stored for possible later use.

**Figure 3:**
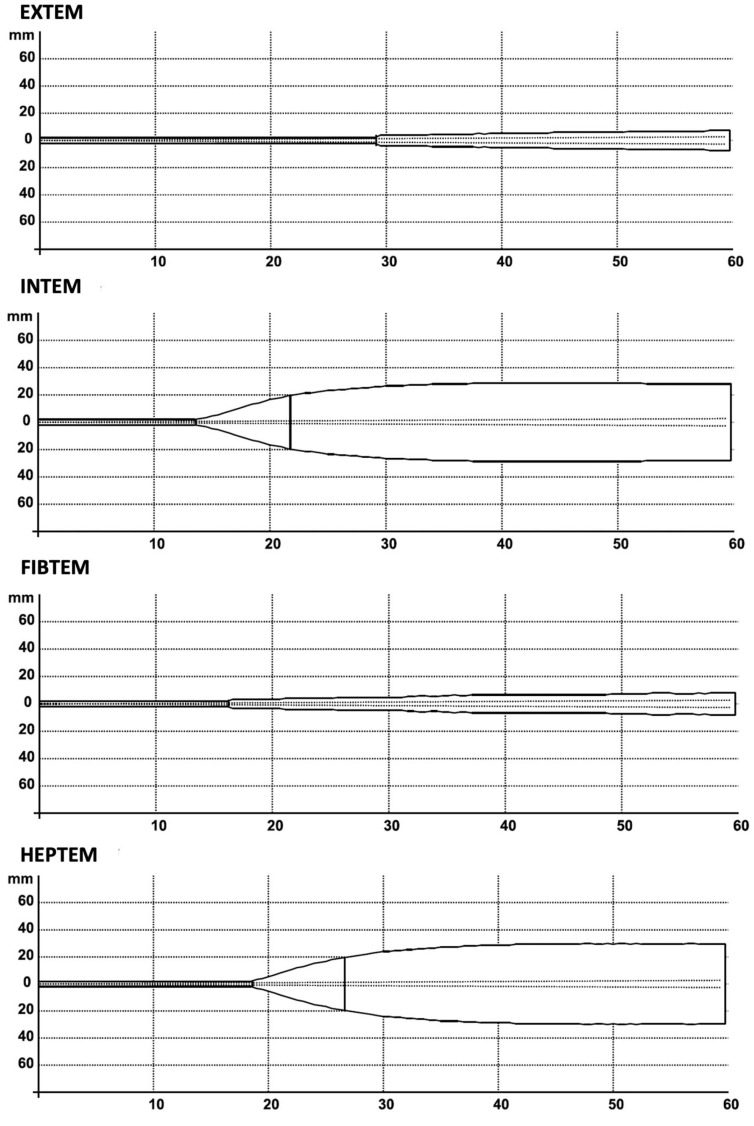
Rotational thromboelastometry (ROTEM) post-surgery.

The patient was transferred to the intensive care unit (ICU) while still intubated and sedated but remained stable with minimal inotropic support (noradrenaline, dobutamine, and levosimendan). The HM3 flow rate was maintained at 2.2 L/min with the pump operating at 4500 rpm. During the first 30 min in the ICU, there was significant blood drainage of 350 mL. Rotational thromboelastometry (ROTEM) revealed hypocoagulability (Figure 3), whereupon we administered blood from the Cell Saver, 6 g of fibrinogen, two units of packed red blood cells, and two units of platelets to the patient. This gradually stabilized the blood drainage while the hemoglobin remained low (89 g/dL). We then administered two more units of packed red blood cells and scheduled the patient for a revision in the operating room 4.5 h after the initial procedure to correct the persistent hemostatic problems. Remarkably, the ACT was 267 with 6 h and 20 min after the discontinuation of argatroban.

**Figure 4:**
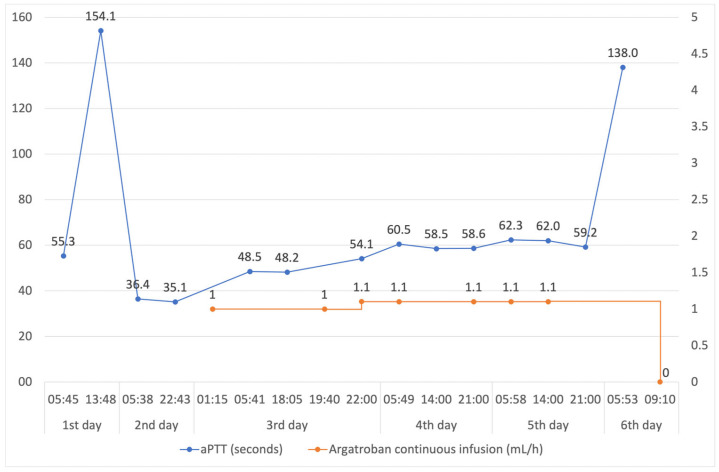
aPTT values on the day of surgery (1st day) and in the following days and argatroban continuous infusion (administration of argatroban on the day of surgery is omitted and is presented in Figure 1).

After revision, both blood drainage and the patient’s clinical condition stabilized. Consequently, we closed the chest 1 day after surgery and reintroduced argatroban at a minimal dose. The argatroban dosage was adjusted according to the activated partial thromboplastin time (aPTT) values (Figure 4). After the discontinuation of sedation, the patient woke up normally so that he could be extubated on the 4th day after surgery. Ventilation and oxygenation remained satisfactory with minimal supplemental oxygen. On the 5th day after surgery, the patient was transferred to the intermediate care unit in an improved and stable clinical condition.

The patient underwent a successful heart transplant 2 months after LVAD implantation.

## 4. Discussion

In this article, we address the significant challenges associated with using argatroban in cardiac surgery for patients with HIT. Our clinical case illustrates many of these difficulties, and our literature review confirms that such issues are not isolated but rather reflect broader concerns in the field.

The timing of LVAD implantation in relation to HIT diagnosis was carefully considered. Thrombocytopenia developed on days 8–9 of hospitalization, with HIT type II promptly confirmed and heparin discontinued. Although in some cases it might be reasonable to delay surgery to allow platelet recovery or explore alternative anticoagulation setups, in our patient this was not a viable option. He was in critical condition with end-stage heart failure, dependent on continuous inotropic support, and clinically deteriorating despite maximal medical therapy. In addition, no viable offers for heart transplantation were available at that time. Waiting posed a significant risk of further hemodynamic decompensation. Therefore, the urgency of LVAD implantation outweighed the potential benefits of postponement. This highlights the need to individualize decisions based on clinical urgency, transplant availability, and hemodynamic stability in HIT-positive patients.

Due to the unavailability of bivalirudin in our center, argatroban was chosen as an alternative anticoagulant for the patient with HIT. Our internal protocol targeted an ACT above 400 s; however, this approach proved inadequate. Despite achieving the target ACT, we observed clot formation in the HLM reservoir. Although a slight drop to 398 s was noted—and prompt adjustments were made—the clots were detected nearly 20 min later. Furthermore, an ACT measurement of 503 s, taken 38 min after discontinuing argatroban, confirmed that the target anticoagulation level was maintained, suggesting that factors beyond the ACT target may contribute to thrombosis.

Several potential explanations exist for these observations. Variability between commercially available ACT assays and limitations in ACT’s ability to fully reflect the anticoagulant effect of argatroban may play a role. Other influencing factors, such as thrombocytopenia, hypothermia, or fibrinogen deficiency, were considered but seem less likely given the patient’s preoperative laboratory values and maintained normothermia. It is also plausible that mechanisms not yet fully described could promote thrombosis during ECC under argatroban anticoagulation [7,22]. Another possible explanation is that critically ill patients with HIT—especially those requiring mechanical circulatory support—may develop a prothrombotic or mixed coagulopathic state independent of the anticoagulant used. This condition may be driven by systemic inflammation, organ dysfunction, and endothelial activation, complicating anticoagulation management even when ACT appears therapeutic [23].

In addition to these biological limitations, ACT monitoring also presents practical challenges. While ACT is widely available and easily used intraoperatively and in catheterization labs, it is often difficult to implement reliably in intensive care settings. This can limit its utility for postoperative anticoagulation monitoring in critically ill patients, particularly when continuous or frequent bedside testing is required [24].

The relatively short ECC duration of 54 min and our extensive experience with LVAD implantation allowed us to mitigate the immediate risks despite the clot formation. The early anastomosis of the outflow graft to the aorta, as described in previous studies, helped minimize stasis time [25,26,27]. The use of TEE further ensured that no thrombi were present in the heart or device components, avoiding potentially fatal complications [14].

Consistently with earlier reports [16,17,18,19], we observed a prolonged normalization of clotting times—extending beyond 6 h—and significant postoperative bleeding that necessitated surgical revision. This discrepancy between the expected pharmacokinetic decline based on argatroban’s half-life and the actual clinical findings, which could not be explained by impaired liver function, underscores the urgent need for more comprehensive in vivo studies. Such research should aim to better define the pharmacokinetics and pharmacodynamics of argatroban during cardiac surgery, ultimately guiding the development of improved dosing and monitoring protocols.

Overall, our experience combined with the literature review highlights the pressing need for refined anticoagulation strategies and advanced monitoring techniques when using argatroban in high-risk cardiac surgery settings [16,17,19]. Beyond argatroban, our findings underscore the critical need for developing and validating alternative anticoagulation strategies tailored to HIT patients, particularly in urgent surgical contexts. These may include standardized protocols for bivalirudin use, the integration of adjunctive antiplatelet therapies, or novel direct thrombin inhibitors under investigation. Clinical cases such as ours clearly illustrate the importance of continued research and protocol optimization to ensure the safe and effective management of this complex patient population.

## 5. Conclusions

Argatroban, as an anticoagulant for cardiac surgery, should be used with caution due to its unpredictable pharmacokinetics and limited experience. Based on our experience, we recommend a target ACT of more than 500 and frequent monitoring during LVAD implantation when argatroban is used for anticoagulation. In addition, we recommend adapting the surgical approach to shorten the stasis time in the LVAD and minimize ECC duration as much as possible. Importantly, our case illustrates the need not only to optimize existing argatroban protocols but also to invest in the development of alternative anticoagulation strategies for patients with HIT. Further research on the pharmacokinetics of argatroban is essential to establish improved protocols for the administration and monitoring of argatroban during cardiac surgery. Moreover, timing decisions should be guided by patient-specific hemodynamic status and transplant availability, as delaying surgery in HIT patients is not always feasible.

## Data Availability

The raw data supporting the conclusions of this article will be made available by the authors upon request.
